# Natural History of Asymptomatic and Unrepaired Vascular Rings: Is Watchful Waiting a Viable Option? A New Case and Review of Previously Reported Cases

**DOI:** 10.3390/children3040044

**Published:** 2016-12-21

**Authors:** Rohit S. Loomba

**Affiliations:** Department of Cardiology and Department of Cardiothoracic Surgery, Children’s Hospital of Wisconsin, 9000 Wisconsin Avenue, Milwaukee, WI 53226, USA; rloomba@chw.org; Tel.: +1-414-266-2082

**Keywords:** vascular ring, right aortic arch, double aortic arch, asymptomatic, unrepaired, vascular anomaly

## Abstract

Vascular rings are a rare form of congenital heart disease in which abnormal aortic arch anatomy leads to encircling of the esophagus and/or trachea by the aortic vasculature. Symptoms can develop from this and prompt the need for surgery. A natural history study has been done on mildly symptomatic patients but no such study has been done on asymptomatic patients. We present a case report of three children with asymptomatic vascular rings who continue to receive follow-up without intervention and review all published cases of asymptomatic or unrepaired vascular rings. Clinical observation of asymptomatic and mildly symptomatic vascular rings, regardless of aortic arch anatomy, seems to be a safe approach. Children with mild symptoms almost invariably seem to have resolution of their symptoms by four years of age.

## 1. Introduction

Vascular rings consist of aortic arch anomalies in which the aorta and its branching vessels encircle the trachea, esophagus, or both structures: these include double aortic arch, right aortic arch with aberrant left subclavian artery, and left aortic arch with aberrant right subclavian artery among others. The first two variations are the most common and combined represent approximately 90% of all vascular rings. Vascular rings are deemed complete if they involve both the trachea and esophagus or incomplete if they only involve one of the two. These anomalies represent 1% to 3% of all congenital heart disease, although this figure may be an underestimate of their true prevalence [1,2].

Manifestations of vascular rings become apparent when compression of the trachea or esophagus become significant and include stridor, wheezing, cough, dyspnea, dysphagia, and recurrent respiratory infections. Generally, patients with double aortic arch begin to demonstrate symptoms in the first few months of life which is in contrast to patients with a right aortic arch and an aberrant left subclavian artery who tend to develop symptoms in their school-age years [3,4,5,6]. Some patients with any variety of vascular ring may be diagnosed incidentally when undergoing imaging for other indications.

Presence of symptoms should prompt consideration of surgical correction of the vascular ring which can vary based on the specific aortic arch anatomy and associated lesions. While those with moderate to severe symptoms should certainly be intervened upon, those with mild symptoms or the absence of symptoms represent a group of patients in which the need for intervention may be less clear as data on the natural history of unrepaired and mildly symptomatic vascular rings is scarce. We present three cases of mildly symptomatic or asymptomatic vascular rings as well as a review of reported cases of asymptomatic or unrepaired vascular rings.

## 2. Case Report 1

**Figure 1:**
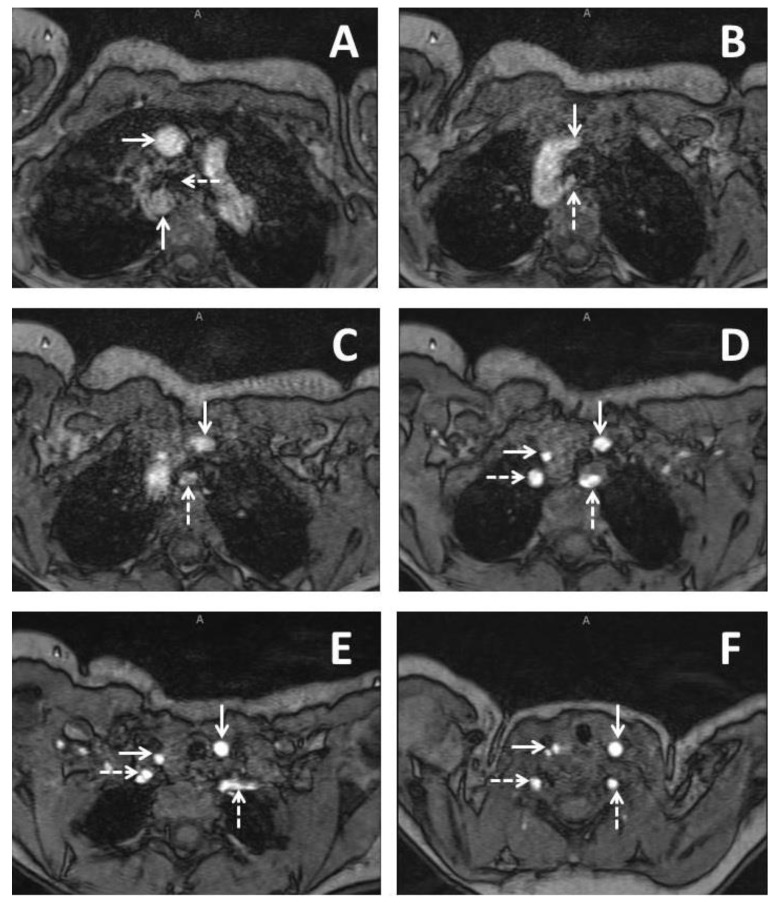
Imaging from a three months old baby girl with DiGeorge syndrome who had some issues with feeding. Series of axial slices from magnetic resonance imaging presented in an inferior to superior progression. Panel **A** demonstrates a right sided aortic arch with the ascending aorta (solid horizontal arrow) coursing anterior to the right bronchus (dashed horizontal arrow) and the descending aorta (solid vertical arrow) coursing immediately posterior to the right bronchus; Panel **B** demonstrates the left common carotid (solid vertical arrow) as the first branching head and neck vessel and arises from the ascending aorta. An aberrant left subclavian artery (dashed vertical arrow) is seen as the last branching head and neck vessel and arises from the descending aorta; Panel **C** demonstrates the course of the left common carotid (solid vertical arrow) and aberrant left subclavian artery (dashed vertical arrow); Panel **D** demonstrates bifurcation of the brachiocephalic trunk into the right common carotid (solid horizontal arrow) and the right subclavian artery (dashed horizontal arrow). The left common carotid (solid vertical arrow) and the left subclavian artery (dashed vertical arrow) are seen demonstrated as well; Panel **E** demonstrates the right vertebral artery arising from the right subclavian artery (dashed horizontal arrow). The right common carotid (solid horizontal line), left common carotid (solid vertical arrow, and aberrant left subclavian (dashed vertical arrow) are also demonstrated; Panel **F** demonstrates early bifurcation of the right common carotid into the right internal and external carotid arteries (solid horizontal arrow). The right vertebral artery (dashed horizontal arrow), the left common carotid (solid vertical arrow), and the left vertebral artery are also demonstrated (dashed vertical arrow).

A three months old baby girl with a history of DiGeorge syndrome and hard palate paralysis was admitted to the general pediatrics service for abnormal movements concerning for seizures. Electroencephalography was consistent with seizures and she subsequently underwent magnetic resonance imaging of her brain as well as magnetic resonance angiography of her head and neck vessels. The resulting imaging demonstrated a right aortic arch with an aberrant left subclavian artery, consistent with a vascular ring (Figure 1). She was referred to the cardiology clinic for further management. At the time of her presentation to the cardiology clinic the young girl was feeding well with appropriate weight gain and had no appreciable stridor or cough. At the time of publication, the young girl is 28 months old and continues to thrive without any surgical intervention for her vascular ring. She tolerates oral feeds without any difficulty and continues to have no stridor, no chronic cough, and no recurrent upper respiratory infections.

## 3. Case Report 2

**Figure 2:**
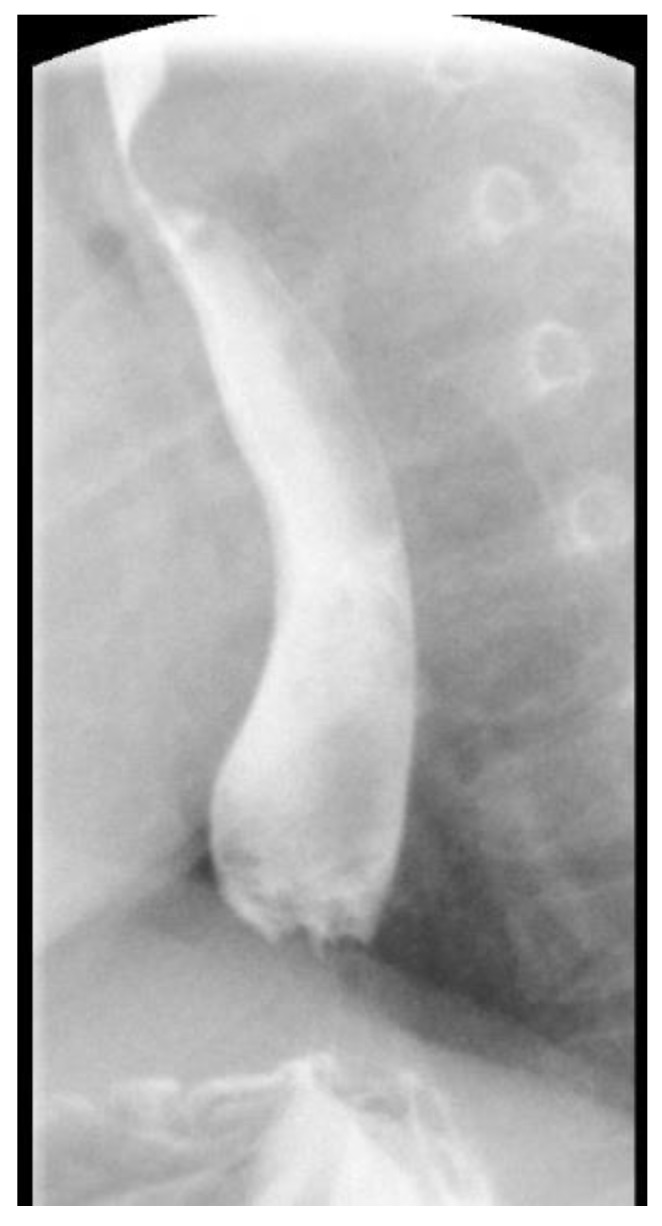
A lateral projection of an upper gastrointestinal imaging study in a 21 months old girl with posterior fossa malformations–hemangiomas–arterial anomalies–cardiac defects–eye abnormalities–sternal cleft and supraumbilical raphe (PHACES) syndrome who had a right aortic arch which demonstrates posterior impingement of the esophagus.

A now 21 months old baby girl was seen prenatally at 22 weeks gestation for concern of an aortic arch anomaly and was noted to have a right aortic arch at that time. The baby and her twin were delivered at 29 weeks gestational age due to severe maternal preeclampsia at which point the baby was also noted to have multiple hemangiomas and was diagnosed with posterior fossa malformations–hemangiomas–arterial anomalies–cardiac defects–eye abnormalities–sternal cleft and supraumbilical raphe (PHACES) Syndrome. She was discharged home from the neonatal intensive care unit (NICU) at eight weeks of life and seen in the cardiology clinic. At seven months of age she underwent magnetic resonance imaging and angiography of her brain as well as head and neck vessels which demonstrated a right aortic arch with aberrant left subclavian artery, consistent with a vascular ring. At 13 months of age she developed a “gurgling sound” during feeds but with absence of choking symptoms and thus underwent upper gastrointestinal imaging which demonstrated posterior impingement on the esophagus which was also consistent with the vascular ring (Figure 2). She did not have any respiratory symptoms. At 21 months of age she remains unintervened upon continues to gain weight well and other than the persistence of gurgling sounds with feeds has done well without any respiratory symptoms. She is able to feed on both solid and soft foods alike.

## 4. Case Report 3

A two years old girl with a history of double inlet left ventricle and D-transposition of the great arteries with ventricular septal defect status post arterial switch procedure underwent a catheterization at the age of two years old for evaluation of pulmonary hypertension. While previous echocardiograms had demonstrated a right sided aortic arch, the anatomy of the branching vessels remained unclear. Catheterization demonstrated a right aortic arch with aberrant left subclavian artery consistent with a vascular ring. She is nearly three years old and continues to demonstrate no symptoms of a vascular ring without any intervention on her aortic arch or branching vessels.

## 5. Discussion

We present three cases of a patient who was incidentally found to have a vascular ring. Because of the absence of symptoms they continue to be clinically observed without any intervention. Vascular rings are a rare form of congenital heart disease and data regarding asymptomatic and unrepaired vascular rings is limited. This is not surprising as vascular rings are generally only diagnosed when they are symptomatic and thus require intervention. The lack of data on the remainder of these rings, however, may cause for uncertainty amongst care providers as to how to manage patients with mild symptoms or the absence of symptoms. Data available on these subsets of patients, particular those that are asymptomatic, are often not found in pediatric cardiology literature but fetal literature.

Natural history data for symptomatic but unrepaired vascular rings comes from a single study published by Godtfredsen et al. in 1977. This study consists of 11 patients with a mean age of diagnosis of 13 months (range of 2–48 months). Three of these patients had a double aortic arch, three had a right aortic arch with aberrant left subclavian artery, and five had left aortic arch with aberrant right subclavian artery. At the time of presentation, seven patients had stridor, six a history of repeat upper respiratory infection, and nine had dysphagia. All of these patients were deemed to have mild symptoms and thus were observed and received median follow-up of seven years at the time of the study. Of these patients, six required medical treatment for their symptoms and eventually had complete resolution of their symptoms as did the remaining five patients. No patients remained symptomatic after four years of age and there were no deaths during the reported follow-up period [7]. No such formal natural history evaluation of asymptomatic vascular rings has been published to date.

An extensive review of the literature yields a total of 58 asymptomatic or unrepaired vascular rings documented over the course of 18 studies, including the current study [4,7,8,9,10,11,12,13,14,15,16,17,18,19,20,21,22]. Table 1 summarizes the characteristics of the individual cases while Table 2 summarizes the overall characteristics of the cohort. Of these 58 cases, 25 (43%) were prenatally diagnosed and 33 (57%) were postnatally diagnosed. Prenatal diagnosis was generally made with appreciation of U-shaped configuration of the aorta and the ductus arteriosus with the trachea located between these two vessels in the 3-vessel and trachea view during fetal ultrasonography. This in contrast to the normal V-shaped configuration which is appreciated in normal aortic arch anatomy in which the right arm of the V is formed by the aortic arch and the left arm of the V is formed by the main pulmonary artery. The trachea, in these cases, is situated to the right of the aortic arch [23]. Three-dimensional power Doppler ultrasound has also been demonstrated to be helpful in the prenatal diagnosis of aortic arch anomalies [21]. Prenatal diagnosis was made at a median gestational age of 21 weeks (range 19–37) and was confirmed, in all cases, either by magnetic resonance imaging or computed tomography imaging of the aortic arch after birth [10,14,19,20,21]. Reported follow-up of these patients after birth ranged from six months to 56 months at the time of their publication. After birth, two patients did develop symptoms with one developing mild stridor at three years of age and the other developing stridor and dysphagia which resolved by eight months of age. There were no deaths reported.

For the 33 postnatally diagnosed cases, the median age of diagnosis was 13 months of age (range 2–876 months). Arch anatomy amongst all 56 cases consisted predominantly of a right aortic arch with aberrant left subclavian artery (47%) which is consistent with the generally accepted view that this anatomy lends itself to a looser vascular ring. Interestingly, double aortic arch was present in 21% of the cases despite being a complete form of a vascular ring and being the most common arch anatomy in which vascular rings are symptomatic. It is also of note that five of the 12 (42%) cases of double aortic arch were diagnosed in adults ranging from 30 to 73 years of age.

A total of 19 associated cardiac anomalies were present among 13 (23%) of all patients with the most frequent being ventricular septal defect which was noted in five (9%) patients. Tetralogy of Fallot was the second most frequent and was noted in two (4%) patients. This finding is consistent with previous studies that have demonstrated an association between vascular rings and Tetralogy of Fallot and ventricular septal defects [5]. A total of 15 extracardiac anomalies were present among 11 patients (20%) with esophageal atresia being the most common, being noted in two (4% patients). These also included a single patient with 22q11 deletion, a single patient with PHACES syndrome, and a single patient with Trisomy 21. Compared to previous studies the incidence of 22q11 in this cohort is lower [24].

The median age at most recent follow-up reported for the patients in this cohort was 50 months (range 4–828). This figure represents data that was available for 25 (43%) of included patients. While all studies describe follow-up, many do not explicitly state the precise duration of follow-up at the time of publication. The natural history study by Godtfredsen et al. has the most robust reporting of follow-up and amongst its 11 patients had a median follow-up duration of seven years. In the documented follow-up, symptoms were present in 15 (27%) patients and consisted of stridor in 11 patients, dysphagia in 10 patients, recurrent infection in six patients, and cough in one patient. Symptoms had resolved by the time of the individual publications in all but two (4%) patients. For children with vascular rings and mild symptoms it appears that resolution of symptoms is frequent and clinical observation is ample in this population, regardless of the specific aortic arch anatomy [7,10,20].

## 6. Conclusions

Clinical follow-up of patients with asymptomatic and mildly symptomatic vascular rings, regardless of the specific aortic arch anatomy, is reasonable and safe. Mild symptoms tend to improve as the child gets older and often completely resolve by the age of four years.

## Figures and Tables

**Table 1:** Individual patient characteristics.

Study	Case	Prenatal Diagnosis	Gestational Age of Diagnosis If Prenatal (Weeks)	Age at Diagnosis If Postnatal (Months)	Arch Anatomy	Cardiac Anomalies	Extracardiac Anomalies	Age at Most Recent Follow-up (Months)	Presence of Any Symptoms
Current study	1	No	--	3	RAA, ALSA	None	DiGeorge, scoliosis	27	No
	2	Yes	29	--	RAA, ALSA	None	PHACES	3	No
	3	No	--	28	RAA, ALSA	DILV, DTGA	None	34	No
Patel et al. [9]	4	Yes	31	--	RAA, LPDA, ALSA	None	None	72	No
Bakker et al. [8]	5	No	--	--	LAA, ARSA	None	None	--	No
	6	No	--	--	LAA, ARSA	None	None	--	No
	7	No	--	--	LAA, ARSA	None	None	--	No
	8	No	--	--	LAA, ARSA	None	None	--	Stridor
Bonnard et al. [4]	9	No	--	--	LAA, ARSA	None	EA	--	No
	10	No	--	--	LAA, ARSA	None	None	--	No
	11	No	--	--	LAA, ARSA	None	None	--	No
	12	No	--	--	LAA, ARSA	None	None	--	No
Hsu et al. [10]	13	Yes	20		RAA, ALSA	None	None	42	No
	14	Yes	22	--	RAA, ALSA	None	None	18	No
	15	Yes	21	--	RAA, ALSA	None	None	9	Stridor, dysphagia
Philip et al. [22]	16	No	--	168	RAA, LDAo	None	--	--	No
	17	No	--	192	RAA, LDAo, ALSA	None	--	--	No
	18	No	--	12	RAA, LDAo, ALSA	ASD, PAPVC	--	--	No
	19	No	--	96	LAA, RDAo	DORV, AVC, LI	--	--	No
Kafka et al. [11]	20	No	--	360	DAA	None	--	--	No
Kindler et al. [12]	21	No	--	873	DAA	CAD	DVT, stroke	--	No
Lee et al. [13]	22	No	--	804	DAA	None	Multinodal goiter	828	Cough, stridor
Miranda et al. [14]	23	Yes	--	--	RAA, ALSA	TOF	--	--	No
	24	Yes	--	--	DAA	TOF	--	--	No
Jan et al. [15]	25	No	--	84	LAA, ARSA	None	None	--	No
	26	No	--	72	RAA, ALSA	None	None	--	No
Seo et al. 2010 [19]	27	Yes	23	--	DAA	VSD	None	4	No
Seo et al. 2011 [16]	28	No	--	432	DAA	None	None	--	No
Vallette et al. [17]	29	No	--	--	--	--	--	--	No
Ikenouchi et al. [18]	30	No	--	876	DAA	MI	None	--	No
Chaoui et al. [21]	31	Yes	23	--	RAA, ALSA	None	None	--	No
Galindo et al. [20]	32	Yes	19	--	DAA	None	None	56	No
	33	Yes	20		RAA, ALSA	None	None	55	No
	34	Yes	20	--	RAA, ALSA	None	None	52	No
	35	Yes	20	--	RAA, ALSA	None	None	51	No
	36	Yes	31	--	RAA, ALSA	None	EA	38	No
	37	Yes	19	--	RAA, ALSA	None	None	28	No
	38	Yes	20	--	RAA, ALSA	None	None	25	No
	39	Yes	20	--	RAA, ALSA	None	None	17	No
	40	Yes	20	--	RAA, ALSA	None	None	15	No
	41	Yes	34	--	RAA, ALSA	VSD	None	35	No
	42	Yes	21	--	RAA, ALSA	None	single umbilical artery	49	No
	43	Yes	22	--	DAA	None	None	37	No
	44	Yes	20	--	RAA, ALSA	None	None	12	No
	45	Yes	24	--	RAA, ALSA	None	Trisomy 21	12	Stridor
	46	Yes	21	--	RAA, ALSA	None	None	13	No
	47	Yes	37	--	RAA, ALSA	None	Hydronephrosis	12	No
Godtfredsen et al. [7]	48	No	(study mean of 13 months)	--	DAA	VSD	HP, CP	(study median of 97 months)	Stridor, dysphagia, recurrent infection
	49	No	--	--	DAA	None	None	--	Recurrent infection
	50	No	--	--	DAA	None	None	--	Stridor, dysphagia, recurrent infection
	51	No	--	--	RAA, ALSA	None	Cognitive delay	--	Stridor, dysphagia, recurrent infection
	52	No	--	--	RAA, ALSA	VSD	None	--	Stridor, recurrent infection
	53	No	--	--	RAA, ALSA	None	None	--	Stridor, dysphagia
	54	No	--	--	LAA, ARSA	None	None	--	Dysphagia, stridor
	55	No	--	--	LAA, ARSA	VSD, PS, AS	Cognitive delay	--	Dysphagia
	56	No	--	--	LAA, ARSA	None	None	--	Dysphagia
	57	No	--	--	LAA, ARSA	None	None	--	Dysphagia, recurrent infection
	58	No	--	--	LAA, ARSA	None	None	--	Dysphagia, stridor

LAA = left aortic arch, RAA = right aortic arch, LPDA = left patent ductus arteriosus, ALSA = aberrant left subclavian artery, ARSA = aberrant right subclavian artery, DAA = double aortic arch, LLA = left ligamentum arteriosoum, REAA = retroesophageal aortic arch, LDAo = left descending aorta, ASD = atrial septal defect, RDAo = right descending aorta, DORV = double outlet right ventricle, AVC = atrioventricular canal, LI = left isomerism, CAD = coronary artery disease, TOF = tetralogy of fallot, VSD = ventricular septal defect, MI = myocardial infarction, PA = pulmonary atresia, RI = right isomerism, EA = esophageal atresia, HP = hypoparathyroidism, CP = cleft palate, PS = pulmonary stenosis, AS = aortic stenosis, DILV = double inlet left ventricle, TGA = transposition of great arteries.

**Table 2:** Summary characteristics for entire cohort.

**Gestational Age of Prenatal Diagnosis (Weeks)**	**Age of Postnatal Diagnosis**
21 (19–37)	13 (2–876)
**Prenatal Diagnosis**	**Age at most recent follow-up (months)**
Yes- 25	***(Available for 25 patients, 43%)***
No- 33	50 (4–828)
***Associated cardiac anomalies***	***Associated extracardiac anomalies***
***(In a total of 14 patients, 24%)***	***(In a total of 12 patients, 21%)***
ASD- 1	DiGeorge- 1
PAPVC- 1	Scoliosis- 1
DORV- 1	Esophageal atresia- 2
AVC- 1	Trisomy 21- 1
LI- 1	Hydronephrosis- 1
RI- 1	Single umbilical artery- 1
CAD- 1	Multinodal goiter- 1
TOF- 2	DVT- 1
VSD- 5	Stroke- 1
MI- 1	Cognitive delay- 2
PS-1	Cleft palate- 1
AS- 1	Hypoparathyroidism- 1
DILV- 1	PHACES syndrome- 1
TGA- 1	
***Arch anatomy***	***Presence of symptoms***
RAA, ALSA- 27 (47%)	***(Present in 15 patients, 26%)***
LAA, ARSA- 14 (24%)	Stridor- 11
DAA- 12 (21%)	Dysphagia- 10
RAA, LDAo- 1 (2%)	Recurrent infection- 6
RAA, LDAo, ALSA- 2 (4%)	Cough- 1
LAA, RDAo- 1 (2%)	
Data not available- 1 (2%)	

LAA = left aortic arch, RAA = right aortic arch, LPDA = left patent ductus arteriosus, ALSA = aberrant left subclavian artery, ARSA = aberrant right subclavian artery, DAA = double aortic arch, LLA = left ligamentum arteriosoum, REAA = retroesophageal aortic arch, LDAo = left descending aorta, ASD = atrial septal defect, RDAo = right descending aorta, DORV = double outlet right ventricle, AVC = atrioventricular canal, LI = left isomerism, CAD = coronary artery disease, TOF= tetralogy of fallot, VSD = ventricular septal defect, MI = myocardial infarction, PA = pulmonary atresia, RI = right isomerism, EA = esophageal atresia, HP = hypoparathyroidism, CP = cleft palate, PS = pulmonary stenosis, AS = aortic stenosis.
